# Heme Oxygenase-1 Protects Retinal Endothelial Cells against High Glucose- and Oxidative/Nitrosative Stress-Induced Toxicity

**DOI:** 10.1371/journal.pone.0042428

**Published:** 2012-08-03

**Authors:** Áurea F. Castilho, Célia A. Aveleira, Ermelindo C. Leal, Núria F. Simões, Carolina R. Fernandes, Rita I. Meirinhos, Filipa I. Baptista, António F. Ambrósio

**Affiliations:** Centre of Ophthalmology and Vision Sciences, IBILI, Faculty of Medicine, University of Coimbra, Coimbra, Portugal; Children’s Hospital Boston, United States of America

## Abstract

Diabetic retinopathy is a leading cause of visual loss and blindness, characterized by microvascular dysfunction. Hyperglycemia is considered the major pathogenic factor for the development of diabetic retinopathy and is associated with increased oxidative/nitrosative stress in the retina. Since heme oxygenase-1 (HO-1) is an enzyme with antioxidant and protective properties, we investigated the potential protective role of HO-1 in retinal endothelial cells exposed to high glucose and oxidative/nitrosative stress conditions. Retinal endothelial cells were exposed to elevated glucose, nitric oxide (NO) and hydrogen peroxide (H_2_O_2_). Cell viability and apoptosis were assessed by MTT assay, Hoechst staining, TUNEL assay and Annexin V labeling. The production of reactive oxygen species (ROS) was detected by the oxidation of 2′,7′-dichlorodihydrofluorescein diacetate. The content of HO-1 was assessed by immunobloting and immunofluorescence. HO activity was determined by bilirubin production. Long-term exposure (7 days) of retinal endothelial cells to elevated glucose decreased cell viability and had no effect on HO-1 content. However, a short-time exposure (24 h) to elevated glucose did not alter cell viability, but increased both the levels of intracellular ROS and HO-1 content. Moreover, the inhibition of HO with SnPPIX unmasked the toxic effect of high glucose and revealed the protection conferred by HO-1. Oxidative/nitrosative stress conditions increased cell death and HO-1 protein levels. These effects of elevated glucose and HO inhibition on cell death were confirmed in primary endothelial cells (HUVECs). When cells were exposed to oxidative/nitrosative stress conditions there was also an increase in retinal endothelial cell death and HO-1 content. The inhibition of HO enhanced ROS production and the toxic effect induced by exposure to H_2_O_2_ and NOC-18 (NO donor). Overexpression of HO-1 prevented the toxic effect induced by H_2_O_2_ and NOC-18. In conclusion, HO-1 exerts a protective effect in retinal endothelial cells exposed to hyperglycemic and oxidative/nitrosative stress conditions.

## Introduction

Diabetic retinopathy is a common complication of diabetes *mellitus* and is a leading cause of vision loss and blindness in working-age adults in developed countries [1]. Diabetic retinopathy is considered a microvascular disease. Retinal microvascular dysfunction is clinically characterized by the increase in blood-retinal barrier permeability, capillary occlusion, the formation of microaneurysms, cotton-wool spots and lipid exudates, and the appearance of hemorrhages. At the later stages, macular edema and neovascularization may also occur. Hyperglycemia is considered the main factor for the development of vascular complications in diabetes [2], triggering the cascade of metabolic and biochemical changes occurring in this pathology. Several evidences also demonstrate that hyperglycemia is associated with an increase in both vascular and neural cell death in the retina [3], [4]. Biochemical alterations induced by hyperglycemia also lead to an increase in oxidative and nitrosative stress [5], [6]. Indeed, a large body of evidence supports the idea that the increase in oxidative stress in retinal microvasculature is a key factor for the development of diabetic retinopathy [7], [8], [9].

The heme oxygenase (HO) family is composed of three isozymes: HO-1, the inducible form, and HO-2 and HO-3, which are constitutively expressed [10], [11]. These enzymes catalyze the degradation of heme groups into equimolar amounts of biliverdin, ferrous iron and carbon monoxide (CO). HO-1 is considered an antioxidant and cytoprotective enzyme [12]. HO-1 antisense and knockout studies, as well as clinical investigations of HO-1 promoter polymorphisms, have clearly shown that HO-1 assumes a central role in cellular antioxidant defenses, especially in vascular protection [13], [14]. Biliverdin and its metabolic product, bilirubin, exert strong antioxidant effects at physiological plasma concentrations. Bilirubin also has cytoprotective and anti-inflammatory functions [15], [16]. Moreover, HO-1 derived CO has anti-apoptotic and cytoprotective actions and modulates the expression of genes that regulate vasoconstriction and inflammatory processes [17], [18].

Concerning the retina, it is known that HO-1 is expressed by pigmented epithelial cells, photoreceptors, ganglion cells, glial cells and endothelial cells [19], [20], and that HO-1 is increased under stress conditions [21], [22]. It was also reported that HO-1 mRNA is upregulated in the retinas of diabetic rats [19]. The vascular protection conferred by HO-1 has been widely investigated in endothelial cells from several tissues. However, under hyperglycemic conditions, the potential protective role of HO-1 in retinal endothelial cells was not studied yet. Therefore, the aim of this study was to investigate the potential protective role of HO-1 against elevated glucose and oxidative/nitrosative stress-induced retinal endothelial cell degeneration.

## Results

### Effect of Long-term Exposure to High Glucose on Retinal Endothelial Cell Viability and HO-1 Levels

**Figure 1:**
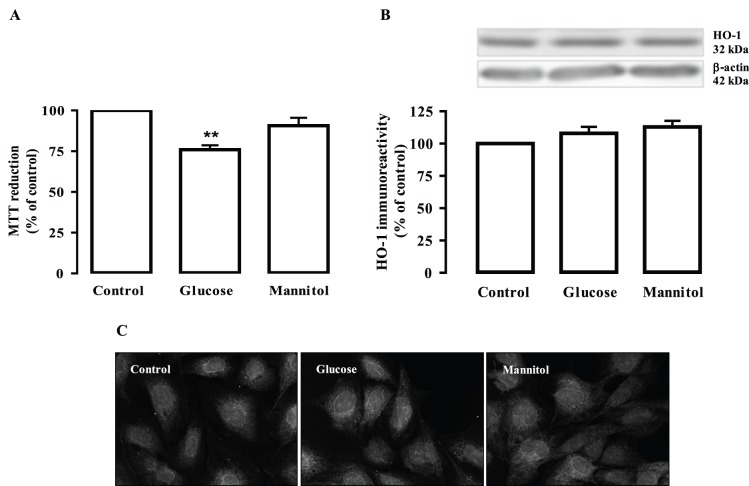
Long-term exposure to high glucose decreases the viability of retinal endothelial cells and does not change HO-1 protein levels. Cells were exposed to 30 mM glucose or mannitol (24.5 mM+5.5 mM glucose; osmotic control) for 7 days. Cell viability was assessed by the MTT reduction assay (A). HO-1 immunoreactivity was analysed by Western blotting (B) and by immunocytochemistry (C). In (B), representative Western blots for HO-1 are presented above the graph. The intensity of the bands was determined by quantitative densitometric analysis. In (C), the representative images were acquired in a confocal microscope (600x magnification). The results represent the mean ± SEM of at least six independent experiments, performed in triplicate in the case of the MTT assay, and are expressed as percentage of control. **p<0.01; significantly different from control as determined by one-way ANOVA followed by *Dunnett’s post test*.

We first evaluated the effect of a long-term exposure of retinal endothelial cells to high glucose (30 mM glucose for 7 days), which mimics chronic hyperglycemic conditions in diabetes, on cell viability, using the MTT reduction assay. Endothelial cells were also exposed to mannitol (24.5 mM+5.5 mM glucose), which was used as an osmotic control. The viability of endothelial cells exposed to high glucose decreased significantly (76.0±2.6% of the control; p<0.01). The exposure to mannitol did not significantly decrease the MTT reduction (Figure 1A).

It is well established that oxidative stress plays a major role in the pathogenesis of diabetic retinopathy, and particularly in retinal endothelial cell dysfunction and death [23], [24], [25]. Since HO-1, an inducible enzyme, has strong antioxidant properties, we evaluated whether HO-1 immunoreactivity could be affected by the long-term exposure to elevated glucose or mannitol, by western blotting and immunocytochemistry. However, the exposure of endothelial cells to high glucose or mannitol, for 7 days, did not alter the HO-1 protein levels (Figure 1B and 1C).

### Effect of Short-term Exposure to High Glucose and Oxidative/nitrosative Stress Conditions on Retinal Endothelial Cell Viability and HO-1 Levels

**Figure 2:**
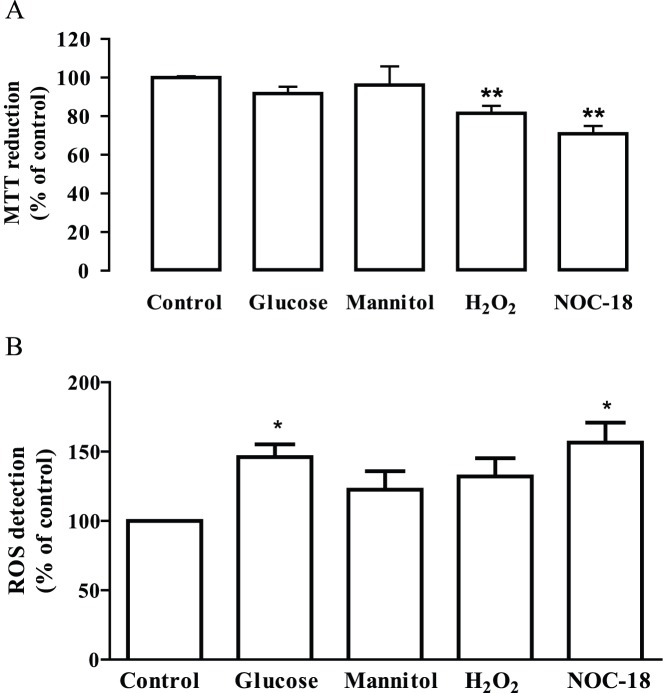
Exposure of retinal endothelial cells to stress conditions decreases cell viability and increases intracellular ROS production. Cells were exposed to 30 mM glucose, mannitol (24.5 mM+5.5 mM glucose), 100 µM H_2_O_2_ or 250 µM NOC-18 for 24 h. Cell viability was assessed by the MTT reduction assay (A). The results represent the mean ± SEM of at least five independent experiments, performed in triplicate, and are expressed as percentage of control. The production of intracellular ROS was assessed by the oxidation of 2′,7′-dichlorodihydrofluorescein diacetate to the fluorescent 2′,7′-dichlorofluorescein. The results represent the mean ± SEM of six independent experiments and are expressed as percentage of control of the ratio arbitrary units/total protein. *p<0.05 and **p<0.01; significantly different from control, as determined by one-way ANOVA followed by *Dunnett’s post test*.

After evaluating the effect of long-term exposure to high glucose on endothelial cell viability, we also analyzed the effect of a short-term exposure to high glucose, as well as the effect of exposure to H_2_O_2_ or NOC-18 (nitric oxide donor), which are known to induce oxidative/nitrosative stress conditions, on cell viability. Cells were exposed to 30 mM glucose (mannitol was used again as an osmotic control), 100 µM H_2_O_2_ or 250 µM NOC-18 for 24 h, and cell viability was evaluated by the MTT reduction assay. Unlike long-term exposure, the exposure to high glucose for 24 h, and to mannitol, did not decrease cell viability (Figure 2A). However, the exposure of endothelial cells to H_2_O_2_ or NOC-18 significantly decreased cell viability (81.5±3.9% and 71±3.9% of the control, respectively; p<0.01) (Figure 2A). We also measured the generation of intracellular reactive oxygen species (ROS) in cells exposed to elevated glucose, mannitol, H_2_O_2_ and NOC-18 for 24 h (Figure 2B). Since ROS measurement was performed at 24 h time point, we must keep in mind that these results do not translate correctly the intracellular formation of ROS along 24 h. Moreover, it is known that H_2_O_2_ is rapidly degraded, and so, concerning the exposure to H_2_O_2_, cells are acutely exposed to high levels of oxidative stress during just the first minutes (and not during 24 h), which is enough to trigger cell death. In the case of NOC-18, the release of NO is continuous, meaning that cells are continuously exposed to nitrosative stress. The levels of intracellular ROS detected at 24 h time point in cells exposed to elevated glucose and NOC-18 increased comparing to control cells. The levels of ROS detected in cells exposed to H_2_O_2_ were not significantly increased despite a trend toward an increase (Figure 2B).

**Figure 3:**
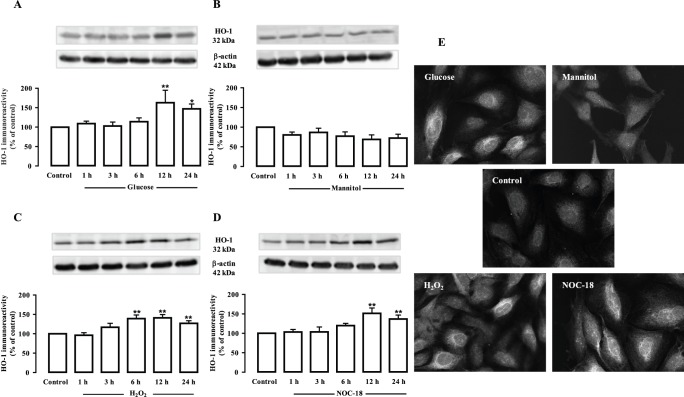
Exposure to high glucose, H_2_O_2_ or NOC-18 increases HO-1 protein levels in retinal endothelial cells. Cells were exposed to 30 mM glucose (A), mannitol (24.5 mM+5.5 mM glucose) (B), 100 µM H_2_O_2_ (C) or 250 µM NOC-18 (D) for 1, 3, 6, 12 or 24 h. HO-1 immunoreactivity was analysed by Western blotting (A-D) and by immunocytochemistry (E). Representative Western blots for HO-1 are presented above the graphs. The intensity of the bands was determined by quantitative densitometric analysis. The images in (E) were acquired in a confocal microscope (600x magnification). The results represent the mean ± SEM of at least three independent experiments, and are expressed as percentage of control. *p<0.05, **p<0.01; significantly different from control as determined by one-way ANOVA followed by *Dunnett’s post test*.

**Figure 4:**
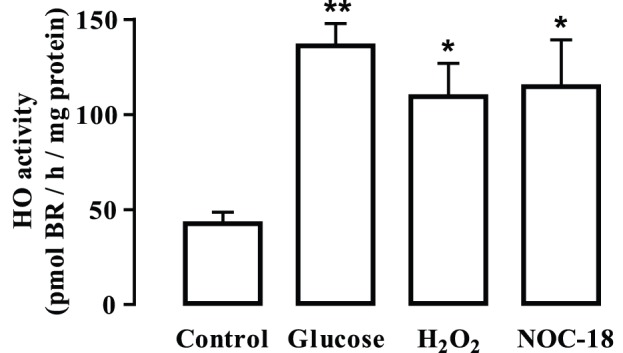
Exposure to high glucose, H_2_O_2_ or NOC-18 increases HO-1 activity in retinal endothelial cells. Cells were exposed to 30 mM glucose, 100 µM H_2_O_2_ or 250 µM NOC-18 for 24 h. Enzymatic activity was determined spectrophotometrically, by measuring the formation of bilirubin (BR). Data are presented as mean ± SEM of at least five independent experiments and are expressed as picomol of BR per hour and per mg of total protein. *p<0.05, **p<0.01; significantly different from control as determined by one-way ANOVA followed by *Dunnett’s post test*.

Since cells treated with elevated glucose, H_2_O_2_ or NOC-18 are under oxidative/nitrosative stress conditions, which in turn could upregulate the expression of HO-1, the immunoreactivity of HO-1 was analyzed in endothelial cells exposed to 30 mM glucose, 24.5 mM mannitol, 100 µM H_2_O_2_ or 250 µM NOC-18 for 1, 3, 6, 12 and 24 h. The exposure to high glucose increased HO-1 levels in a time-dependent manner (62.9±32.0% and 46.9±12.7% increase at 12 and 24 h exposure; p<0.01 and p<0.05; respectively) (Figure 3A). On the other hand, mannitol did not induce any significant changes on HO-1 levels comparing to control (Figure 3B). The exposure to H_2_O_2_ induced a significant increase in HO-1 immunoreactivity (39.1±9.5%, 41.2±8.2% and 26.9±6.6% increase compared to control after 6, 12 and 24 h, respectively; p<0.01) (Figure 3C). Similarly, the NO donor, NOC-18, induced a significant increase in HO-1 protein levels (51.4±13.9% and 36.4±10.1% increase compared to control at 12 and 24 h, respectively; p<0.01) (Figure 3D). By immunocytochemistry, we also found that the immunoreactivity of HO-1 increased when endothelial cells were exposed to high glucose, H_2_O_2_ and NOC-18 for 24 h (Figure 3E). The immunoreactivity observed in cells exposed to mannitol for 24 h was similar to control (Figure 3E). Accompanying the rise in HO-1 levels, there was also an increase in the enzymatic activity of HO (measured in pmol bilirubin/h/mg protein) in cells exposed to high glucose, H_2_O_2_ or NOC-18 for 24 h (Figure 4).

### Effect of HO-1 Inhibition on Viability of Endothelial Cells Exposed to High Glucose and Oxidative/nitrosative Stress Conditions

**Figure 5:**
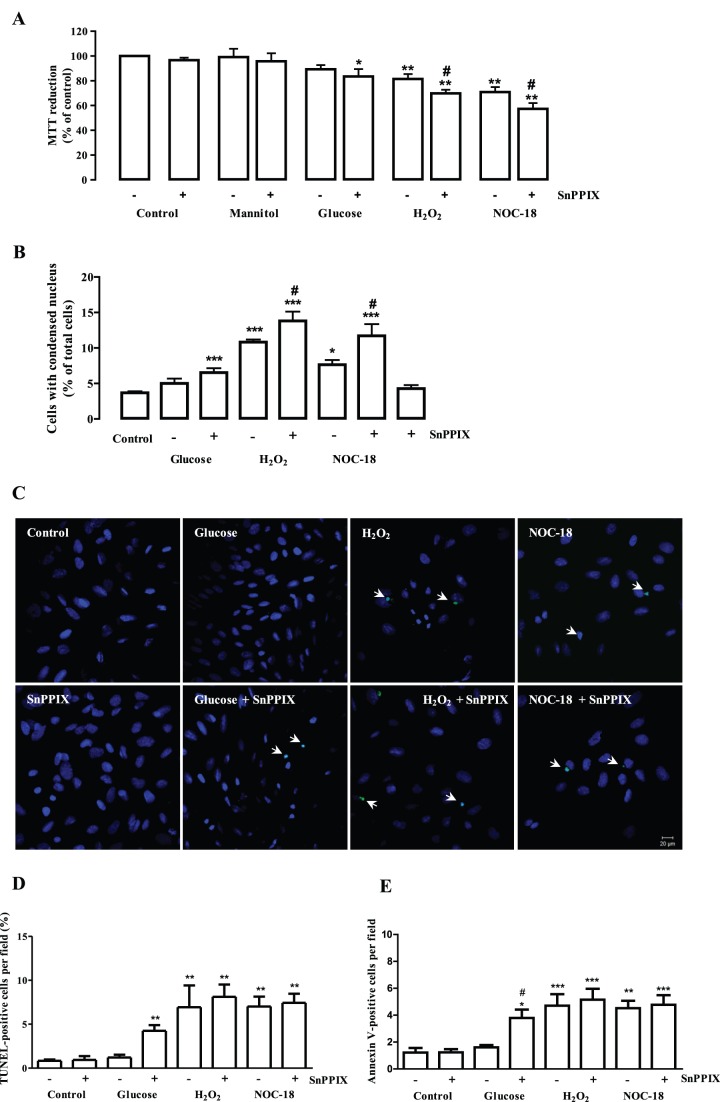
HO-1 inhibition enhances hyperglycemic toxicity and endothelial cell susceptibility to H_2_O_2_ or NOC-18. Cells were exposed to 30 mM glucose, mannitol (24.5 mM+5.5 mM glucose), 100 µM H_2_O_2_ or 250 µM NOC-18 for 24 h, in the absence or in the presence of SnPPIX (10 µM), a HO inhibitor. Cell viability was assessed by the MTT reduction assay (A). The results represent the mean ± SEM of at least four independent experiments, performed in triplicate, and are expressed as percentage of control. Cell death was determined by Hoechst staining. Cells with condensed/fragmented nuclei were considered apoptotic (B). The results represent the mean ± SEM of four independent experiments. *p<0.05, **p<0.01, ***p<0.005; significantly different from control (one-way ANOVA followed by *Dunnett’s post test*). #p<0.05; significantly different from a similar condition, but in the absence of SnPPIX (one-way ANOVA followed by *Bonferronís post test*). Apoptotic cells were identified either by TUNEL assay or Annexin V labeling (C–E). The TUNEL staining images were acquired in a confocal microscope (400x magnification, scale bar 20 µm) and show TUNEL staining in green and nuclei staining with DAPI in blue (C). The quantitative results from TUNEL assay represent mean ± SEM of four independent experiments and are presented as percentage of TUNEL-positive cells per field (D). The results from Annexin V labeling represent mean ± SEM of five independent experiments and are presented as Annexin V-positive cells per field (E).

As previously shown in Figure 2, the exposure of endothelial cells to H_2_O_2_ or NOC-18 decreases cell viability. Since HO-1 protein levels and HO activity increased in cells exposed to oxidative/nitrosative stress conditions, as well as in cells treated with high glucose, we evaluated whether the inhibition of HO could further affect the viability of endothelial cells exposed to those conditions. The incubation of retinal endothelial cells with SnPPIX, an HO inhibitor, for 24 h, did not decrease cell viability (Figure 5A) or increased the number of cells with condensed nuclei, as well as TUNEL- and Annexin V-positive cells (Figure 5B–5E and Figure S3). As expected, the exposure to mannitol for 24 h, both in the absence and in the presence of SnPPIX, did not affect cell viability. Interestingly, although the short-term exposure (24 h) to high glucose did not significantly affect cell viability (see Figure 2A), the simultaneous exposure of endothelial cells to elevated glucose and SnPPIX induced a significant reduction in cell viability (83.5±6.0% of the control; p<0.05), an increase in cells with condensed nuclei (6.5±0.6%, comparing to 3.7±0.2% in control; p<0.005) (Figure 5B), in TUNEL-positive cells (4.3±0.6%, comparing to 0.9±0.2% in control) (Figure 5C and 5D) and in Annexin V-positive cells (3.8±0.6 positive cells per field comparing to 1.2±0.3 in control) (Figure 5E and Figure S3).

The cell line used in this study was immortalized using the SV40, which could affect glucose metabolism in these cells. In order to confirm the effect of elevated glucose exposure for 24 h on endothelial cell death, in the presence or absence of HO inhibition, we also performed the TUNEL assay in primary endothelial cells (human umbilical vascular endothelial cells - HUVECs). Similarly to the results obtained with TR-iBRB, the exposure to elevated glucose for 24 h did not increase cell death in HUVECs (Figure S1). However, the exposure to elevated glucose in the presence of the HO inhibitor, SnPPIX, significantly increased the percentage of TUNEL-positive HUVECs (Figure S1).

We have also evaluated the effect of HO inhibition on the production of ROS in retinal endothelial cells exposed to elevated glucose, H_2_O_2_ or NOC-18. In these conditions, the generation of ROS was increased comparing to control cells (Figure S2). The presence of SnPPIX further enhanced the decrease in cell viability induced by exposure to H_2_O_2_ or NOC-18 (69.9±2.9% of control and 57.3±4.7% of control, respectively; p<0.01, Figure 5A), the increase in cells with condensed nuclei (13.8±1.3% and 11.7±1.7% of condensed nuclei, respectively; p<0.01, Figure 5B), in TUNEL-positive cells (7.4±1.0% and 8.1±1.4, comparing to 0.9±0.2% in control, Figure 5C and 5D) and in Annexin V-positive cells (5.2±0.8 and 4.8±0.7 positive cells per field comparing to 1.2±0.3 in control) (Figure 5E and Figure S3).

### Effect of HO-1 Overexpression on Viability of Endothelial Cells Exposed to Oxidative/nitrosative Stress Conditions

**Figure 6:**
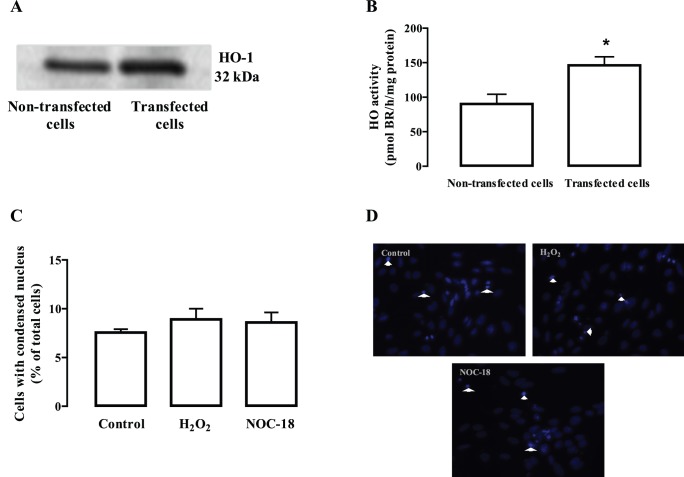
Overexpression of HO-1 protects retinal endothelial cells from the toxic effect of H_2_O_2_ and NOC-18. Cells were transfected with pcDNA3-HO-1 by electroporation. After electroporation, cells were allowed to recover for 24 h and then were exposed to 100 µM H_2_O_2_ or 250 µM NOC-18 for 24 h. Representative Western Blot showing an increase in the protein content of HO-1 in transfected cells (A). Enzymatic activity on electroporated cells was determined spectrophotometrically, by measuring the formation of bilirubin (BR). Data are presented as mean ± SEM of five independent experiments and are expressed as pmol of BR per hour and per mg of total protein (B). *p<0.05; significantly different from non-electroporated cells as determined by *Student’s t test*. Cell death by apoptosis was assessed by Hoechst staining (C, D). The images were acquired in a fluorescence microscope (400x magnification). The results represent the mean ± SEM of at least four independent experiments.

As shown previously, 24 h exposure to H_2_O_2_ or NOC-18, but not to high glucose, decreased endothelial cell viability. Since HO-1 inhibition exacerbates the toxicity in retinal endothelial cells exposed to oxidative/nitrosative stress conditions, which supports a protective role of HO-1, we evaluated the potential protective effect of HO-1 overexpression in endothelial cells exposed to H_2_O_2_ or NOC-18. As can be seen in Figure 6A, the content of HO-1 increased by 25.6±12.5% in cells transfected with pcDNA3-HO-1. In order to confirm whether the protein being expressed was fully functional, we also measured HO activity in cells transfected with pcDNA3-HO-1. In these cells, HO activity increased by 56±12 pmol BR/h/mg protein relatively to non-transfected cells (Figure 6B). In cells overexpressing HO-1, no toxic effect was found when they were exposed to H_2_O_2_ or NOC-18 for 24 h (Figure 6C and 6D), contrarily to what had been found in non-transfected cells (see Figures 2 and 5).

## Discussion

Diabetic retinopathy is characterized by progressive alterations in the retinal microvasculature, and hyperglycemia has been considered the prime triggering factor for the increase in blood-retinal barrier permeability, which is the first signal clinically detected in diabetic retinopathy [26]. A large body of evidence has also demonstrated an increase in reactive oxygen species and NO production in different tissues and cell types during diabetes, or after exposure to high glucose [9], [27], which have been claimed to contribute to the vascular alterations observed in diabetic retinopathy. In fact, oxidative and nitrosative stress have been associated to the increase of apoptosis in retinal endothelial cells exposed to hyperglycemic conditions [8], [24], [25], [28]. Here, we also demonstrate that elevated glucose *per se* induces an increase in the levels of ROS in retinal endothelial cells. Regarding H_2_O_2_, since we measure the levels of ROS production 24 h after adding H_2_O_2_ to the culture media, and H_2_O_2_ is rapidly converted into other species, we might be underestimating the production of ROS when cells are exposed to H_2_O_2_ alone. In fact, the ROS levels measured were not significantly higher in cells exposed to H_2_O_2_ comparing to control. In cells exposed to NOC-18, the ROS levels were significantly increased. As in cells exposed to high glucose, where glucose levels are kept elevated during the 24 h incubation, in cells exposed to NOC-18 the release of NO is continuously maintained. Moreover, the inhibition of HO further increased the levels of ROS in cells exposed to H_2_O_2_ and NOC-18, indicating that when HO is active, it is able to protect cells from excessive production of free radicals.

HO-1 is an enzyme with antiapoptotic and anti-inflammatory properties. Its expression is induced under stress conditions, and it has been reported to be involved in the protection of several cell types [29], [30], [31], [32]. However, very little is known about the behaviour of HO-1 in the retina under hyperglycemic or stress conditions. Therefore, since HO-1 might protect retinal cells, and particularly retinal endothelial cells, against diabetes-induced endothelial cell degeneration, we evaluated its potential protective role in retinal endothelial cells exposed to high glucose and oxidative/nitrosative stress conditions.

We have previously shown that a long-term exposure (7 days) of retinal endothelial cells to elevated glucose increases cell death [33]. Here we also investigated the effect of a short-term exposure (24 h) on endothelial cell death and show that elevated glucose did not significantly affect cell viability. Interestingly, short-term exposures to elevated glucose (12 h and 24 h) induced a significant increase in HO-1 protein levels, which was not detected for longer exposures (7 days). Under prolonged exposures cells might adapt to the environmental conditions and the induction of HO-1 expression is stopped, similarly to what happens under hypoxic conditions [34]. On the other hand, the continuous induction of HO-1 could lead to a local increase of CO or heme, which might be potentially toxic to cells [35].

The exposure to H_2_O_2_ and NOC-18 for 24 h decreased retinal endothelial cell viability, and under these conditions there was an increase in HO-1 protein levels. The increase in HO-1 expression induced by H_2_O_2_ or NO donors was previously shown in several studies and in several cell types, including neurons, epithelial and endothelial cells, but not in retinal endothelial cells [36], [37], [38]. The upregulation of HO-1 content in retinal endothelial cells exposed to elevated glucose (short-term exposure), H_2_O_2_ or NOC-18 was accompanied by an increase in HO activity, which led us to suspect that HO-1 could be exerting protective effects in endothelial cells. In fact, the protective role of HO-1 in retinal endothelial cells was unmasked when the HO inhibitor, SnPPIX, was used. When HO-1 was inhibited, the toxic effect induced by H_2_O_2_ and NOC-18 was potentiated as shown by the results of the MTT assay and Hoechst staining. The inhibition of HO did not significantly increase the percentage of TUNEL- or Annexin V-positive cells in H_2_O_2_- or NOC-18- treated cells, comparing to cells exposed to H_2_O_2_ or NOC-18 in the absence of HO inhibition, but there was a tendency for an increase. However, and more important, because hyperglycemia is a key factor and a trigger of diabetic complications, was, the protection conferred by HO-1 in retinal endothelial cells exposed to elevated glucose (24 h). In this case, the protection was clearly unmasked when HO-1 was inhibited by SnPPIX, since under these conditions there was a significant decrease in endothelial cell viability (MTT assay) and a significant increase in endothelial cell apoptosis (Hoechst nuclear staining, TUNEL assay and Annexin V labeling). The exposure to elevated glucose in the absence of SnPPIX did not significantly affect cell viability or cell death. These results indicate that high glucose is toxic for endothelial cells, not only for longer periods (7 days), but also after 24 h exposure. However, the toxic effect induced by elevated glucose is masked because retinal endothelial cells are able to protect themselves, at least temporarily, through the upregulation of HO-1. For long term-exposures the protective effect of HO-1 appears to be lost. The protective role of HO-1 was also demonstrated when HO-1 was overexpressed in endothelial cells treated with H_2_O_2_ and NOC-18, where it completely prevented the toxic effect induced by these compounds. In vascular smooth muscle cells and in lung fibroblasts transfected with HO-1 there was also a prevention of the toxicity caused by oxidative stress conditions [39], [40]. Moreover, transfection of renal proximal tubular epithelial cells with HO-1 protected cells from cold storage-induced cell injury [41]. This study indicates that HO-1 is upregulated in retinal endothelial cells exposed to hyperglycemic or oxidative/nitrosative stress conditions, and this increase is associated with a protective effect.

In conclusion, HO-1 is able to protect retinal endothelial cells exposed to elevated glucose or oxidative/nitrosative stress conditions, and therefore it may protect the retinal vascular endothelium under diabetic conditions. However, this cytoprotective role of HO-1 appears to be transitory, becoming compromised in longer exposure to elevated glucose. This impairment of the antioxidative defenses in the retina might contribute, in part, to the degenerative processes occurring in diabetic retinopathy.

## Materials and Methods

### Cell Culture

#### TR-iBRB2 cell line culture

A rat retinal endothelial cell line (TR-iBRB2), established from transgenic rats carrying the temperature-sensitive SV-40 large T antigen gene [42], was cultured in flasks in low-glucose DMEM (Invitrogen Corporation, Paisley, UK) supplemented with 10% FBS (Invitrogen), 100 U/ml penicillin G and 0.1 mg/ml streptomycin at 33°C in a humidified atmosphere with 5% CO_2_. For experiments, as previously described by our group, the cells were plated in collagen A (Biochrom AG, Berlin, Germany)-coated dishes or coverslips at a density of 250 cells per cm^2^ and grown to confluence for 7 days [33], [43], [44]. Cells were incubated with 30 mM D-glucose, 24.5 mM D-mannitol (+5.5 mM D-glucose), 100 µM H_2_O_2_ or 250 µM NOC-18 (Alexis Biochemicals, San Diego, USA) for several time periods. These concentrations are the same used in previous reports [33], [43]. The incubation with SnPPIX (10 µM; Tocris, Bristol, UK) was performed 30 min before adding glucose, mannitol H_2_O_2_ or NOC-18.

#### Human umbilical vascular endothelial cell (HUVEC) culture

HUVEC were plated in gelatin coated coveslips at a density of 6,000 cells per cm^2^ and grown in EGM-2 medium (both cells and medium from Lonza, Walkersville, MD, USA) for 2 days to confluence. Cells were incubated with 30 mM D-glucose for 24 h and the incubation with SnPPIX (10 µM; Tocris, Bristol, UK) was performed 30 min before adding glucose.

### Cell Viability and Cell Death Assays

The viability of endothelial cells was assessed by the colorimetric MTT reduction assay [45]. Cells were plated in 12-well plates and treated with high glucose, mannitol, H_2_O_2_, NOC-18 or SnPPIX, according to figure legends. After treatments, the cell medium was removed and replaced with Krebs solution (in mM: 142 NaCl, 4 KCl, 1 MgCl_2_, 10 glucose and 10 HEPES, pH 7.4) containing 0.5 mg/ml MTT for 90 min at 37°C in a humidified atmosphere with 5% CO_2_. Formazan crystals formed were solubilised with 0.04 M HCl in isopropanol. The absorbance was measured at 570 nm, with a reference filter at 620 nm. The results are presented as mean ± SEM and represent the percentage of control (no treatment).

Cells undergoing apoptosis were identified by nuclear morphology after nuclei staining with Hoechst 33342 (Sigma-Aldrich), by TUNEL assay and Annexin V labbeling.

For the Hoechst staining, cells were fixed in methanol:acetone (1∶1) for 10 min and incubated with Hoechst (5 mg/ml) for 5 min. The images were acquired with an inverted fluorescence microscope (DM IRE2, Leica Microsystems, Cambridge, UK). At least a minimum of 400 nuclei from 5–7 random fields were counted. The results are expressed as condensed nuclei in percentage of total nuclei.

DeadEnd Fluorimetric TUNEL system, in which fluorescein-12-dUTP at is catalytically incorporated at 3′-OH DNA ends using recombinant Terminal Deoxynucleotidyl Transferase, was performed according to the manufacturer’s instructions (Promega Corporation, Madison, WI, USA). Cells were also stained with 4′6-diamidino-2-phenylindole (DAPI) to label all nuclei. The images were acquired in a LSM 710 confocal scanning laser microscope (Zeiss, Gottingen, Germany). At least a minimum of 400 nuclei from 8 random fields were counted. The results are expressed as percentage of TUNEL-positive cells.

For Annexin V labeling, cells were washed twice with phosphate-buffered saline (PBS; in mM: 137 NaCl, 2.7 KCl, 1.8 KH_2_PO_4_ and 10 Na_2_HPO_4_, pH 7.4) and once with Annexin V binding buffer (in mM: 10 HEPES pH 7.4, 140 NaCl and 2.5 CaCl_2_). Next, cells were incubated with Annexin V- FITC (BD Pharmingen, San Diego, CA, USA; 1∶20 in binding buffer) for 15 min at room temperature. Cells were washed with Annexin V binding buffer and immediately visualized under the confocal microscope. Five independent experiments were performed and 7–9 fields per coverslip were counted for each condition.

### Detection of Intracellular ROS Generation

The generation of intracellular reactive oxygen species was detected using the indicator 2′,7′-dichlorodihydrofluorescein diacetate (H_2_DCFDA, Invitrogen Corporation, Paisley, UK), a non-fluorescent probe that is rapidly oxidized to the fluorescent 2′,7′-dichlorofluorescein in the presence of intracellular ROS. Cells were plated in 12-well plates and treated with high glucose, mannitol, H_2_O_2_, NOC-18 or SnPPIX, according to figure legends. After treatments, the cell medium was removed and replaced with Dulbecco’s Phosphate-Buffered Saline (DPBS; in mM: 0.9 CaCl_2_, 0.5 MgCl_2_, 2.7 KCl, 1.7 KH_2_PO_4_, 138 NaCl, 8 Na_2_HPO_4_, 5.6 D-Glucose, 0.3 sodium pyruvate; pH 7.4) containing 5 µM H_2_DCFDA for 30 min at 33°C in the dark and in a humidified atmosphere with 5% CO_2_. The solution with the dye was replaced by DPBS and the cells were incubated at 33°C in the dark and in a humidified atmosphere with 5% CO_2_ for 15 min. Cells were lysed with DPBS with 1% TritonX-100 and the fluorescence was measured in a microplate reader (Synergy HT, BioTek Instruments, Winooski, VT, USA) at excitation and emission wavelengths of 485 and 530 nm, respectively. Protein concentration was determined by the BCA colorimetric assay (Pierce, Rockford, USA). The results are expressed as percentage of control of the ratios AU/total protein in each condition.

### Western Blotting

Endothelial cells were lysed with RIPA buffer (in mM: 150 NaCl, 5 EGTA, 1% Triton X-100, 0.5% DOC, 0.1% SDS and 50 Tris-HCl, pH 7.4), supplemented with a protease inhibitor cocktail (Roche, Basel, Switzerland) and 1 mM DTT. Protein concentration was determined by the BCA colorimetric assay. The lysates (30 µg of total protein per lane) were separated by 4–15% sodium dodecyl sulphate-polyacrylamide gel electrophoresis (SDS-PAGE) and transferred to polyvinylidene fluoride (PVDF) membranes (Amersham Biosciences, Uppsala, Sweden). The membranes were blocked with 5% non-fat milk in TBS-T (in mM: 137 NaCl, 20 Tris, pH 7.6, and 0.1% Tween 20) and then incubated with the primary anti-HO-1 (Stressgene Bioreagents, Victoria, Canada; 1∶2,000) and anti-β-actin (Sigma-Aldric; 1∶20,000) antibodies diluted in 1% non-fat milk in TBS-T for 2 h at room temperature. The membranes were incubated with the secondary antibodies conjugated with alkaline phosphatase (Amersham Biosciences; 1∶20,000) diluted in 1% non-fat milk in TBS-T for 1 h at room temperature. The immunoreactive bands were visualized using the enhanced chemifluorescence (ECF) substrate and an imaging system (STORM 860, Molecular Dynamics, Amersham Biosciences). The densitometry of the bands was quantified using the Image Quant 5.0 software (Molecular Dynamics, Amersham Biosciences).

### HO-1 Immunofluorescence

Endothelial cells were cultured on collagen-coated glass coverslips and exposed to high glucose, mannitol, H_2_O_2_ or NOC-18, according to figure legends. After treatments, the cells were washed with PBS and fixed in 4% paraformaldehyde for 10 min at room temperature, and then washed again with PBS containing 0.03% BSA and 0.02% sodium azide (PBS*). After washing, the cells were permeabilized in 1% Triton X-100 in PBS* for 10 min. Blockage was performed with 10% normal goat serum in PBS*. Following blockage, cells were incubated with the primary antibody anti-HO-1 (1∶200, diluted in PBS*) for 1 h at room temperature. After washing, cells were incubated with a goat anti-rabbit Alexa Fluor 488-conjugated secondary antibody (Molecular Probes, Invitrogen Corporation; 1∶250, diluted in PBS*) for 1 h at room temperature. The images were acquired with a confocal microscope (MRC600, BioRad, Watford, UK).

### HO-1 Transfection

The endothelial cells were transiently transfected by electroporation with a plasmid encoding full-length rat HO-1 cDNA under the control of the CMV promoter (pcDNA3/HO-1), which was kindly donated by Dr. Miguel Soares (Instituto Gulbenkian de Ciência, Portugal).

Briefly, 10 µg of plasmid containing HO-1 was mixed with the cell suspension (5.5×10^5^ cells) to a final volume of 400 µl in a 4 mm-gap electroporation cuvette. Cells were electroporated at a capacitance of 760 µF and at 200 V, using a Gene Pulser electroporator (BioRad, Richmond, USA), which gave a pulse length ranging from 35 to 40 ms. The electroporated cells were left in the cuvette on ice for 10 min and then plated at a density of 6,000 cells/cm^2^. The experiments were performed 48 h after transfection.

### HO Enzymatic Activity Determination

The enzymatic activity of HO was determined by the rate of bilirubin production [46]. Endothelial cells were exposed to high glucose, H_2_O_2_ or NOC-18 for 24 h, and after treatments, cells were solubilised in Tris buffer, supplemented with a protease inhibitor cocktail (in mM: 25 Tris-HCl, pH 7.4, and 1 EDTA, 1 EGTA, 1 PMSF and 1 DTT).

Briefly, cellular extracts (250 to 400 µg of total protein) were added to the reaction mixture containing 0.8 mM NADPH, 2 mM glucose-6-phosphate, 0.2 U/ml glucose-6-phosphate dehydrogenase, 20 µM hemin, 2 mg of rat liver cytosol (source of biliverdin reductase), 2 mM MgCl_2_, 50 mM Tris-HCl, pH 7.4. The mixtures (1 ml total volume) were incubated at 37°C for 1 h in the dark and then placed on ice to terminate the reaction. Bilirubin was extracted with chloroform, and bilirubin production was determined by calculating the difference between the absorbance at 464 nm and 530 nm (extinction coefficient for bilirubin: 40 mM^−1^ cm^−1^). HO activity was expressed as pmol of bilirubin formed per hour per mg of total protein.

### Statistical Analysis

The results are expressed as mean ± S.E.M. Differences among experimental groups were analyzed using one-way ANOVA. The significance of differences between groups was assessed by *Dunnett’s* or *Bonferroni’s post hoc* tests, as indicated in figure legends. Significance was defined for a *p* value <0.05. The statistical analysis was performed using Prism 4.0 software (GraphPad Software, San Diego, USA).

## Supporting Information

Figure S1 **HO inhibition increases hyperglycemic toxicity in HUVECs.** Cells were exposed to 30 mM glucose for 24 h, in the absence or in the presence of SnPPIX (10 µM), a HO inhibitor. Apoptotic cells were identified by TUNEL assay. The images were acquired in a confocal microscope (400x magnification, scale bar 20 µm). The results represent the mean ± SEM of four independent experiments and are presented as percentage of TUNEL-positive cells per field. ***p<0.001; significantly different from control, as determined by one-way ANOVA followed by *Dunnett’s post test*.
(EPS)

Figure S2 **Inhibition of HO increases intracellular ROS production in retinal endothelial cells exposed to elevated glucose and stress conditions.** Cells were exposed to 30 mM glucose, mannitol (24.5 mM+5.5 mM glucose), 100 µM H_2_O_2_ or 250 µM NOC-18 for 24 h, in the absence or in the presence of SnPPIX (10 µM), a HO inhibitor. The production of intracellular ROS was assessed by the oxidation of 2′,7′-dichlorodihydrofluorescein diacetate to the fluorescent 2′,7′-dichlorofluorescein. The results represent the mean ± SEM of six independent experiments and are expressed as percentage of control of the ratio arbitrary units/total protein. *p<0.05 and ***p<0.001; significantly different from control, as determined by one-way ANOVA followed by *Dunnett’s post test*. ##p<0.01; significantly different from a similar condition, but in the absence of SnPPIX as determined by one-way ANOVA followed by *Bonferronís post test*).
(EPS)

Figure S3 **Representative images of Annexin V labeling in retinal endothelial cells.** Cells were exposed to 30 mM glucose, 100 µM H_2_O_2_ or 250 µM NOC-18 for 24 h, in the absence or in the presence of SnPPIX (10 µM), a HO inhibitor. The images were acquired in a confocal microscope (400x magnification, scale bar 20 µm) and show Annexin V staining in green merged with phase contrast image.
(EPS)
